# Sexual dysfunction in women with gestational trophoblastic disease: a cross-sectional study in Recife, Pernambuco, Brazil

**DOI:** 10.7717/peerj.21427

**Published:** 2026-09-25

**Authors:** Edinaldo Brito dos Santos, Elias Ferreira de Melo Jr, Josielle Carvalho de Lima, Auro Del Giglio

**Affiliations:** 1 Centro Universitário FMABC, Santo André, São Paulo, Brazil; 2 Faculdade de Medicina, Universidade Federal de Pernambuco, Recife, Pernambuco, Brazil; 3 Faculdade Estácio do Recife, Recife, Pernambuco, Brazil

**Keywords:** Gestational trophoblastic disease, Hydatidiform mole, Sexual dysfunction, Women’s health, Outpatient care

## Abstract

This study aimed to evaluate the prevalence of sexual dysfunction among women diagnosed with gestational trophoblastic disease (GTD) who were undergoing outpatient follow-up at a university hospital. A cross-sectional study was conducted involving 73 women aged ≥ 18 yr. Data were collected using a structured questionnaire addressing sociodemographic and clinical characteristics, in addition to the Sexual Quotient - Female Version (SQ-F) to assess sexual function. Most participants were aged 18–30 yr (58.9%), married (74.0%), and reported using oral contraceptives (52.1%). Based on the total SQ-F score, 63.0% (*n* = 46) of participantsdemonstrated adequate sexual satisfaction. However, 15.0% reported experiencing dyspareunia, and 45.2% reported difficulty maintaining concentration during sexual activity. Sexual dysfunction was significantly associated with smoking (Prevalence ratios (PR) = 2.88; *p* = 0.015) and residence in rural areas (PR = 3.47; *p* = 0.003). Although the overall prevalence of sexual dysfunction was not higher than national estimates, the findings identify specific vulnerabilities that may adversely affect sexual health. These factors should be considered in the comprehensive clinical management of women with GTD.

## Introduction

Gestational trophoblastic disease (GTD) comprises a heterogeneous group of pregnancy-related disorders originating from placental trophoblastic epithelium. According to the International Federation of Gynecology and Obstetrics (FIGO, 2002) classification system, GTD includes a spectrum of conditions ranging from benign hydatidiform moles to malignant neoplasms that require systemic therapy.

Globally, estimated incidence rates range from 1 to 2 cases per 1,000 pregnancies, with higher rates reported in parts of Asia and Latin America. Population-based studies have demonstrated substantial geographic and temporal variability, reflecting differences in diagnostic practices, healthcare access, and epidemiological surveillance systems (Eysbouts et al., 2016; Joneborg et al., 2018).

Evidence-based reviews underscore the importance of understanding the epidemiological distribution of GTD to accurately contextualize its clinical burden and evaluate its reproductive and psychological consequences (Horowitz et al., 2021). The global incidence of GTD varies considerably. In high-income countries, it affects approximately 1 in 1,000–1,500 pregnancies. In contrast, substantially higher rates are reported in low- and middle-income regions, particularly in parts of Asia and Latin America. In Brazil, the estimated incidence of molar pregnancy ranges from 1 in 200 to 400 pregnancies, highlighting GTD as a significant public health concern in this setting.

Standard management of GTD consists of early diagnosis, prompt uterine evacuation, and meticulous post-molar surveillance through serial measurements of serum human chorionic gonadotropin (hCG). These therapeutic measures are intended to optimize prognosis, preserve reproductive potential, and maintain long-term quality of life. Nevertheless, despite the high cure rates associated with GTD, its diagnosis and prolonged surveillance may exert a considerable psychological and emotional burden. Women frequently report anxiety, depressive symptoms, fear of recurrence, and uncertainty regarding future fertility. Although treatment and follow-up are typically conducted in an outpatient setting, the surveillance phase often entails repeated hospital visits, intensive hCG monitoring, mandatory contraceptive use, and adherence to strict medical recommendations. These demands may disrupt daily activities and impose social or family-related constraints, thereby negatively affecting overall well-being.

While the clinical presentation and therapeutic management of GTD are well established, the impact of GTD management on female sexual function remains insufficiently investigated. Emerging evidence indicates that both the diagnosis and management of GTD may increase psychological vulnerability, thereby adversely affecting sexual well-being and overall quality of life. International studies have consistently documented the substantial psychosocial burden associated with GTD. For instance, Stafford et al. (2011) reported clinically significant levels of depression (22%) and anxiety (26%) among women diagnosed with GTD, with 52% of patients experiencing sexual dysfunction. These findings highlight the presence of persistent psychological morbidity that may exceed that observed in the general population.

Similarly, Di Mattei et al. (2021) emphasized the scarcity of comprehensive data addressing psychological, sexual, and fertility-related concerns in women with GTD. Although most participants reported satisfactory partner relationships, diminished sexual desire was frequently described. Blok et al. (2022) further demonstrated that women with GTD experience elevated levels of distress, anxiety, and depressive symptoms during the early phase following diagnosis, resulting in a marked negative impact on psychological well-being. Collectively, these findings underscore that, beyond its physical consequences, GTD exerts a significant and multifaceted effect on women’s psychological and sexual health.

Studies from high-income countries generally report relatively low incidence rates, ranging from approximately 1 case per 1,000 to 1,500 pregnancies. In contrast, reports from Asian and Latin American countries describe substantially higher rates, with estimates ranging from 1 case per 12 to 1 per 500 pregnancies.

In Brazil, national data indicate that molar pregnancy occurs in approximately 1 in 200–400 pregnancies, representing a higher incidence than that reported in most developed countries (Brazilian Federation of Gynecology and Obstetrics Associations FEBRASGO, 2021).

## Materials & Methods

### Study design and setting

This cross-sectional descriptive study with an exploratory quantitative approach was conducted at the Gynecology and Obstetrics Outpatient Clinic of the Hospital das Clínicas, Federal University of Pernambuco (HC/UFPE), located in Recife, Pernambuco, Brazil. It is a regional referral center for the management of GTD in northeastern Brazil.

### Participants

Women diagnosed with GTD and undergoing outpatient follow-up were considered eligible. A total of 105 women were screened, and 32 were excluded based on predefined criteria. Exclusion criteria comprised uncontrolled severe psychiatric disorders (*e.g.*, active psychosis or acute mood episodes requiring immediate intervention), hospitalization within the preceding 30 days, and cognitive impairment precluding questionnaire completion. Women receiving active chemotherapy for GTD were also excluded to restrict the sample to those in the surveillance phase of treatment.

Inclusion criteria were as follows: confirmed diagnosis of GTD, age ≥18 yrs, and self-reported sexual activity within the previous six months.

### Clinical and histopathological classification

Histopathological diagnosis was established following uterine evacuation and classified as complete or partial hydatidiform mole according to standard pathological criteria. All patients subsequently underwent outpatient follow-up with serial serum human chorionic gonadotropin (hCG) measurements in accordance with institutional protocols.

The diagnosis of gestational trophoblastic neoplasia (GTN) was based on criteria established by the International Federation of Gynecology and Obstetrics. Diagnostic criteria included plateauing or rising hCG levels during follow-up, histological evidence of myometrial invasion, or other clinical indicators of disease progression. Invasive moles were classified as a subtype of GTN and were not categorized as partial or complete hydatidiform moles.

Only women in the surveillance phase of GTD management were eligible. Patients undergoing active chemotherapy for GTN at the time of recruitment were excluded to ensure sample homogeneity.

### Recruitment procedures and potential confounders

Participants were recruited consecutively during their initial post-diagnostic follow-up consultations at the GTD outpatient clinic. Eligible women were approached individually in a private setting by a trained research nurse, who explained the study objectives and procedures. Only women meeting all inclusion criteria and not receiving chemotherapy were invited to participate.

Written informed consent was obtained prior to enrollment. Data collection consisted of a structured interview and completion of validated questionnaires, requiring approximately 20–25 min. The interviewer remained available to clarify questions while ensuring privacy and minimizing external influence.

To enhance methodological rigor, clinical and psychosocial variables with potential confounding effects were documented. These included hormonal status (*e.g.*, contraceptive use and menstrual patterns) and self-reported psychological or psychiatric history. These variables were considered in the analytical model to assess their influence on the associations investigated.

### Sample size

A convenience sample of women receiving outpatient care for GTD was obtained. The final sample size reflected the limited number of eligible patients treated at this referral center and the sensitive nature of the study topic, which may have influenced participation.

### Data collection

Data were collected between September and December. Participants were recruited while awaiting their scheduled medical consultations. Each data collection session lasted approximately 20–25 min and was conducted by a trained female researcher.

The data collection instrument comprised two components. The first was a structured questionnaire developed by the principal investigator to obtain sociodemographic, clinical, obstetric, and lifestyle information. The second component was the Sexual Quotient-Female Version (SQ-F), a validated self-report instrument designed to assess sexual function in Brazilian women. The SQ-F was developed by the ProSex Study Program at the Institute of Psychiatry of the University of São Paulo Medical School. The instrument evaluates domains including sexual desire, arousal, lubrication, orgasm, comfort, and satisfaction across the female sexual response cycle.

Additional clinical data were retrieved from patients’ electronic medical records using the AGHUx system.

### Data analysis

Data were entered into Microsoft Excel and analyzed using SPSS software (version 21). Categorical variables were summarized as absolute and relative frequencies. Associations between variables were assessed using the chi-square test or Fisher’s exact test, as appropriate. PRs and corresponding 95% confidence intervals (95% CIs) were calculated.

Variables with *p* < 0.20 in bivariate analyses were included in a Poisson regression model with robust variance to estimated adjusted PR (aPRs) and 95% CIs.

Model diagnostics were performed to assess adequacy. Overdispersion was evaluated, and the dispersion parameter was approximately 1, indicating no evidence of overdispersion. Multicollinearity was assessed using variance inflation factors, all of which were <2, suggesting the absence of significant collinearity. Model performance was further evaluated using a receiver operating characteristic (ROC) curve.

Regarding missing data, completeness of the analytical dataset was verified. Missing data accounted for <5% and did not affect key variables; therefore, no imputation procedures were applied. Cases with incomplete responses on primary variables were excluded using listwise deletion.

### Ethical considerations

The study was approved by the Research Ethics Committees of Centro Universitário FMABC and the Hospital das Clínicas da Universidade Federal de Pernambuco (CAAE: 35405820.7.0000.0082).

## Results

### Sociodemographic characteristics

Participants ranged in age from 18 to 50 yr, with the majority aged 18–30 yr (*n* = 43, 58.9%). Most were born in rural areas or in other Brazilian states (*n* = 39, 53.7%) and resided in the metropolitan region of Recife (*n* = 31, 42.5%). The majority were married (*n* = 54, 74.0%), had completed secondary education (*n* = 43, 58.9%), self-identified as mixed race (*n* = 40, 54.8%), and were employed at the time of the study (*n* = 41,  56.2%; Table 1).

**Table 1:** Socio demographic characteristics of women undergoing outpatient follow-up for gestational trophoblastic disease. Recife, Pernambuco, Brazil.

**Evaluated factor**	**n**	**%**	*p*-value
**Sex**			
-Feminine	73	100	–
**Gender**			
-Cis gender Woman	73	100	–
**Age**			
–18 to 30 years old	43	58.9	
–31 to 40 years old	22	30.01	<0.001^1^
–41 to 50 years old	8	11.0	
**Place of birth**			
–Recife	12	16.4	
–Metropolitan region of Recife	22	30.1	<0.001^1^
–Interior/Other state	39	53.7	
**Place of residence**			
–Recife	12	16.4	
–Recife Metropolitan Area	31	42.5	<0.001^1^
–Interior/Other state	30	41.1	
**Marital status**			
–Married	54	74.0	
–Single	17	23.3	<0.001^1^
–Divorced/separated	2	2.7	
**Education level**			
–Primary education	21	28.8	
–High school	43	58.9	<0.001^1^
–Higher education/Postgraduate	9	12.3	
**Self-reported race/color**			
-Mixed-race	40	54.8	
-White	14	19.2	
-Black	12	16.4	<0.001^1^
-Asian	7	9.6	
**Employment status**			
–Employed	41	56.2	
–Unemployed	29	39.7	<0.001^1^
–Neverworked	3	4.1	

**Notes.**

1 *p*-value from Chi-square test for proportion comparison.

Source: Prepared by the authors.

### Clinical and epidemiological profile

Most participants had at least one child (*n* = 43, 58.9%), with one child being the most common parity (*n* = 32, 44.2%). Regarding gravidity, 30.1%(*n* = 22) reported one pregnancy and 30.1%(*n* = 22) reported two pregnancies. Vaginal delivery was the most frequent mode of childbirth (*n* = 39, 53.4%). Additionally, 74.3%(*n* = 54) reported at least one miscarriage (Table 2).

**Table 2:** Distribution of clinical and epidemiological characteristics of women receiving outpatient follow-up for gestational trophoblastic disease. Recife/PE, 2023.

**Variable**	**n**	**%**	*p*-value
**Has children**			
Yes	43	58.9	0.128^1^
Not	30	41.1	
**Number of children**			
1 child	19	44.2	
2 children	15	34.9	0.171^1^
3 or more children	9	20.9	
**Number of pregnancies**			
1 pregnancy	22	30.1	
2 pregnancies	22	30.1	0.375^1^
3 pregnancies	14	19.2	
4 or more pregnancies	15	20.6	
**Number of deliveries**			
1 delivery	19	44.2	
2 deliveries	15	34.9	0.171^1^
3 or more deliveries	9	20.9	
**Type of delivery**			
Cesarean section	28	38.4	<0.001^1^
Vaginal delivery	39	53.4	
Other	6	8.2	
**Regular use of medication**			
Yes	50	68.5	0.002^1^
Not	23	31.5	
**Smoking status**			
Smoker	2	2.7	<0.001^1^
Non-smoker	71	97.3	

**Notes.**

1 *p*-value from Chi-squaretest for proportion comparison.

Source: Prepared by the authors.

The majority reported regular medication use (*n* = 50.68.5%) and were nonsmokers (*n* = 71.97.3%), with 95.9% (*n* = 70) indicating no prior history of smoking. No statistically significant differences were observed for the variables “having children” (*p* = 0.128), “number of children” (*p* = 0.171), or “number of pregnancies” (*p* = 0.375), suggesting a balanced distribution across groups.

Family history of cancer was the most frequently reported comorbidity (*n* = 36, 49.3%), followed by systemic hypertension (*n* = 4, 5.5%; *p* = 0.907). Only one participant reported a family history of GTD.

Regarding contraceptive use, oral contraceptives were the most commonly reported method (*n* = 38, 52.1%), followed by injectable contraceptives (*n* = 23, 31.5%) and condoms (*n* = 9, 12.3%). No significant difference in oral contraceptive use was observed between women with and without sexual dysfunction (*p* = 0.725; Table 3).

**Table 3:** Distribution of contraceptive methods used by women in outpatient follow-up for gestational trophoblastic disease.

**Contraceptive method**			*p*-value
	**Yes**	**Not**	
Condom	9 (12.3%)	64 (87.7%)	<0.001
Píl Birth control pill	38 (52.1%)	35 (47.9%)	0.725
Calendar method (table)	0 (0.0%)	73 (100.0%)	–
Withdrawal	1 (1.4%)	72 (98.6%)	<0.001
Patch	0 (0.0%)	73 (100.0%)	–
IUD	0 (0.0%)	73 (100.0%)	–
Injectable contraceptive	23 (31.5%)	50 (68.5%)	0.002
Others	1 (1.4%)	72 (98.6%)	<0.001

**Notes.**

1 *p*-value from Chi-square test for proportion comparison.

Source: prepared by the authors.

### Histopathological diagnosis and clinical classification

Histopathological examination following uterine evacuation identified hydatidiform mole as the predominant initial diagnosis. At the time of referral, most cases were classified as hydatidiform mole without specification of subtype (*n* = 64, 87.7%). A smaller proportion was histologically classified as complete mole (*n* = 5, 6.8%) or partial mole (*n* = 1, 1.4%). One case (1.4%) was diagnosed as hydropic degeneration (Table 4).

**Table 4:** Distribution of histopathological diagnosis after uterine evacuation.

**Evaluated factor**	**n**	**%**	***p***-value
**Postoperative anatomopathological** ** diagnosis**			
Complete	5	6.8	
Hydatidiform	64	87.7	
Partial hydatidiform	1	1.4	<0.001
Invasive	2	2.7	
Partial	1	1.4	

**Notes.**

a *p*-value from Chi-squaretest for proportion comparison.

Source: prepared by the authors.

During outpatient follow-up, two patients (2.7%) met diagnostic criteria established by the International Federation of Gynecology and Obstetrics for GTN and were classified as having invasive moles. Invasive mole was considered a clinical progression of GTD rather than a histopathological subtype of hydatidiform mole.

### Sexual function

Assessment using the SQ-F questionnaire revealed that 52.1%(*n* = 38) reported never or rarely experiencing pain during vaginal penetration. Spontaneous sexual thoughts or fantasies were reported by 46.6%(*n* = 34), and 63.0%(*n* = 46) indicated adequate sexual desire.

Most participants reported positive arousal in response to foreplay (*n* = 61, 83.6%), and 64.4%(*n* = 47) reported adequate vaginal lubrication. Increased arousal in response to partner arousal was reported by 78.1%(*n* = 57), and 76.8%(*n* = 56) indicated sufficient vaginal relaxation for penetration.

Furthermore, 45.2%(*n* = 33) reported being fully focused during sexual intercourse, 63.0%(*n* = 46) reported achieving orgasm, and 71.3%(*n* = 52) reported satisfaction sufficient to motivate future sexual activity (Table 5).

**Table 5:** Evaluation of sexual Quotient-Female (QS-F) questionnaire items in women under outpatient treatment for gestational trophoblastic disease –recife, PE.

		**Response to item**	
**Evaluated Item**	**0 and 1 - Never/rarely**	**2 and 3 - Sometimes/50%** **of the time**	**4 and 5- Most of the** **time/always**
1. Do you spontaneously think about sex, remember sex, or imagine having sex?	19 (26.0%)	34 (46.6%)	20 (27.4%)
2. Is your interest in sex enough for you to engage in sexual activity willingly?	9 (12.3%)	18 (24.7%)	46 (63.0%)
3. Do foreplay (caresses, kisses, hugs, *etc.*) encourage you to continue the sexual activity?	6 (8.2%)	6 (8.2%)	61 (83.6%)
4. Do you usually get lubricated (wet) during sexual intercourse?	8 (10.9%)	18 (24.7%)	47 (64.4%)
5. During sexual intercourse, as your partner’s excitement increases, do you also feel more stimulated for sex?	6 (8.2%)	10 (13.7%)	57 (78.1%)
6. During sexual intercourse, do you relax your vagina enough to facilitate penile penetration?	5 (6.8%)	12 (16.4%)	56 (76.8%)
7. Do you usually feel pain during sexual intercourse when the penis penetrates your vagina?	38 (52.1%)	24 (32.9%)	11 (15.0%)
8. Do you manage to stay focused (without distraction) during sexual intercourse?	17 (23.3%)	23 (31.5%)	33 (45.2%)
9. Can you reach orgasm (sexual pleasure) during the sexual intercourse you have?	7 (9.6%)	20 (27.4%)	46 (63.0%)
10. Does the level of satisfaction you achieve from sexual intercourse make you want to have sex again, on other days?	5 (6.8%)	16 (21.9%)	52 (71.3%)

**Notes.**

Source: Abdo (2009).

### Associations with sexual dysfunction

Table 6 presents the distribution of sexual dysfunction, categorized according to SF-Q scores as “Sexual Dysfunction” (0–60) and “No Sexual Dysfunction” (61–100), stratified by sociodemographic characteristics. No statistically significant associations were observed (*p* > 0.05), indicating that sexual dysfunction was evenly distributed across demographic groups.

**Table 6:** Association between the presence of sexual dysfunction measured by the Female Sexual Quotient (FSQ) score and sociodemographic characteristics of women with gestational trophoblastic disease under outpatient treatment.

**Variable**	**FSQ score ≤ 60 *n* (%)**	**FSQ score > 60 *n* (%)**	**PR (95% CI)**	*p*-value
Age (years)				
18–30	10 (23.3)	33 (76.7)	1.00	–
31–40	6 (27.3)	16 (72.7)	1.17 (0.49–2.80)	0.938^1^
41–50	2 (25.0)	6 (75.0)	1.08 (0.29–4.01)	
City of birth				
Recife	3 (25.0)	9 (75.0)	1.83 (0.44–7.72)	
Metropolitan region of Recife	3 (13.6)	19 (86.4)	1.00	0.329^1^
Countryside/Other state	12 (30.8)	27 (69.2)	2.26 (0.71–7.14)	
City of residence				
Recife	5 (41.7)	7 (58.3)	3.23 (1.03–10.03)	
Metropolitan region of Recife	4 (12.9)	27 (87.1)	1.00	0.099^1^
Countryside/Other state	9 (30.0)	21 (70.0)	2.33 (0.80–6.75)	
Marital status				
Married	11 (20.4)	43 (79.6)	1.00	–
Single	6 (35.3)	11 (64.7)	1.73 (0.75–3.98)	0.245^2^
Separated/Divorced	1 (50.0)	1 (50.0)	2.45 (0.56–10.81)	
Educational level				
Elementary education	7 (33.3)	14 (66.7)	1.79 (0.75–4.28)	
High school	8 (18.6)	35 (81.4)	1.00	0.356^1^
Higher/Postgraduate	3 (33.3)	6 (66.7)	1.79 (0.59–5.47)	
Self-declared race/ethnicity				
Mixed	8 (20.0)	32 (80.0)	1.00	–
White	4 (28.6)	10 (71.4)	1.43 (0.51–4.02)	0.722^2^
Black	4 (33.3)	8 (66.7)	1.67 (0.61–4.59)	
Asian	2 (28.6)	5 (71.4)	1.43 (0.38–5.38)	
Employment status				
Currently employed	8 (19.5)	33 (80.5)	1.00	–
Unemployed	9 (31.0)	20 (69.0)	1.59 (0.70–3.63)	0.431^2^
Never worked	1 (33.3)	2 (66.7)	1.71 (0.31–9.51)	

**Notes.**

PR Prevalence Ratio CI Confidence Interval

1 *p*-value from the Chi-square test for independence.

2 *p*-value from Fisher’s Exact test.

Source: Developed by the authors.

Similarly, clinical and epidemiological variables were not significantly associated with sexual dysfunction (*p* > 0.05; Table 7).

**Table 7:** Distribution of sexual dysfunction according to clinical and epidemiological characteristics of women with gestational trophoblastic disease under outpatient treatment.

Variable	FSQ score ≤ 60 *n* (%)	FSQ score > 60 *n* (%)	PR (95% CI)	*p*-value
Has children				
Yes	13 (30.2)	30 (69.8)	1.81 (0.72–4.55)	0.186^1^
Number of children				
1 child	5 (26.3)	14 (73.7)	1.00	–
2 children	5 (33.3)	10 (66.7)	1.27 (0.45–3.58)	
3 or more children	3 (33.3)	6 (66.7)	1.27 (0.39–4.17)	0.915^2^
Number of pregnancies				
1 pregnancy	4 (18.2)	18 (81.8)	1.27 (0.27–6.05)	
2 pregnancies	6 (27.3)	16 (72.7)	1.91 (0.45–8.17)	
3 pregnancies	2 (14.3)	12 (85.7)	1.00	–
4 or more pregnancies	5 (16.7)	25 (83.3)	0.67 (0.47–3.34)	0.401^2^
Number of deliveries				
1 delivery	5 (26.3)	14 (73.7)	1.58 (0.53–4.74)	
2 deliveries	5 (33.3)	10 (66.7)	2.00 (0.68–5.85)	0.884^1^
3 or more deliveries	3 (33.3)	6 (66.7)	2.00 (0.59–6.79)	
Type of delivery				
Cesarean section	3 (27.3)	8 (72.7)	1.00	–
Vaginal delivery	10 (34.5)	19 (65.5)	1.26 (0.43–3.75)	
1 Cesarean + 1 vaginal	0 (0.0)	2 (100.0)	–	
2 Cesarean + 3 vaginal	0 (0.0)	1 (100.0)	–	1.000^2^
Regular use of medication				
Yes	10 (20.0)	40 (80.0)	1.00	–
No	8 (34.8)	15 (65.2)	1.74 (0.79–3.82)	0.173^1^
Smoking				
Yes	2 (100.0)	0 (0.0)	–	0.058^2^
No	16 (22.5)	55 (77.5)	1.00	–
Former smoker				
Yes	2 (66.7)	1 (33.3)	2.92 (1.18–7.24)	0.148^2^
No	16 (22.9)	54 (77.1)	1.00	–

**Notes.**

PR Prevalence Ratio CI Confidence Interval

1 *p*-value from the Chi-square test for independence.

2 *p*-value from Fisher’s Exact test.

Source: Developed by the authors.

### Final poisson regression model

Variables with *p* ≤ 0.20 in the bivariate analysis (including residence location and smoking status) were entered into the multivariate Poisson regression model with robust variance.

In the final adjusted model, two variables remained independently associated with sexual dysfunction (*p* ≤ 0.05): residence in a rural area or another state (PR = 3.47; 95% CI [1.51–7.97]; *p* = 0.003) and current or past smoking (PR = 2.88; 95% CI [1.23–6.74]; *p* = 0.015; Table 8).

**Table 8:** Final Poisson model adjustment for estimating the risk of developing Null/Regular sexual dysfunction on the QS-F.

**Evaluated factors**	**Evaluated factors**	**Evaluated factors**	**Evaluated factors**
**Place of residence**			
Recife	2.06	0.74–5.76	0.168^1^
Recife Metropolitan Area	1.00	–	–
Country side/Otherstate	3.47	1.51–7.97	0.003^1^
**Smoking**			
Yes	2.88	1.23–6.74	0.015^1^
No	1.00	–	–

**Notes.**

PR Prevalence Ratio CI Confidence Interval.

1 Wald test *p*-value.

Source: Prepared by the authors.

### Predictive model accuracy

Figure 1 presents the ROC curve for the final regression model. The area under the curve (AUC) was 0.899 (*p* < 0.001; 95% CI [0.811–0.987]); however, this result should be interpreted with caution, as it reflects model performance within this sample only.

**Figure 1:**
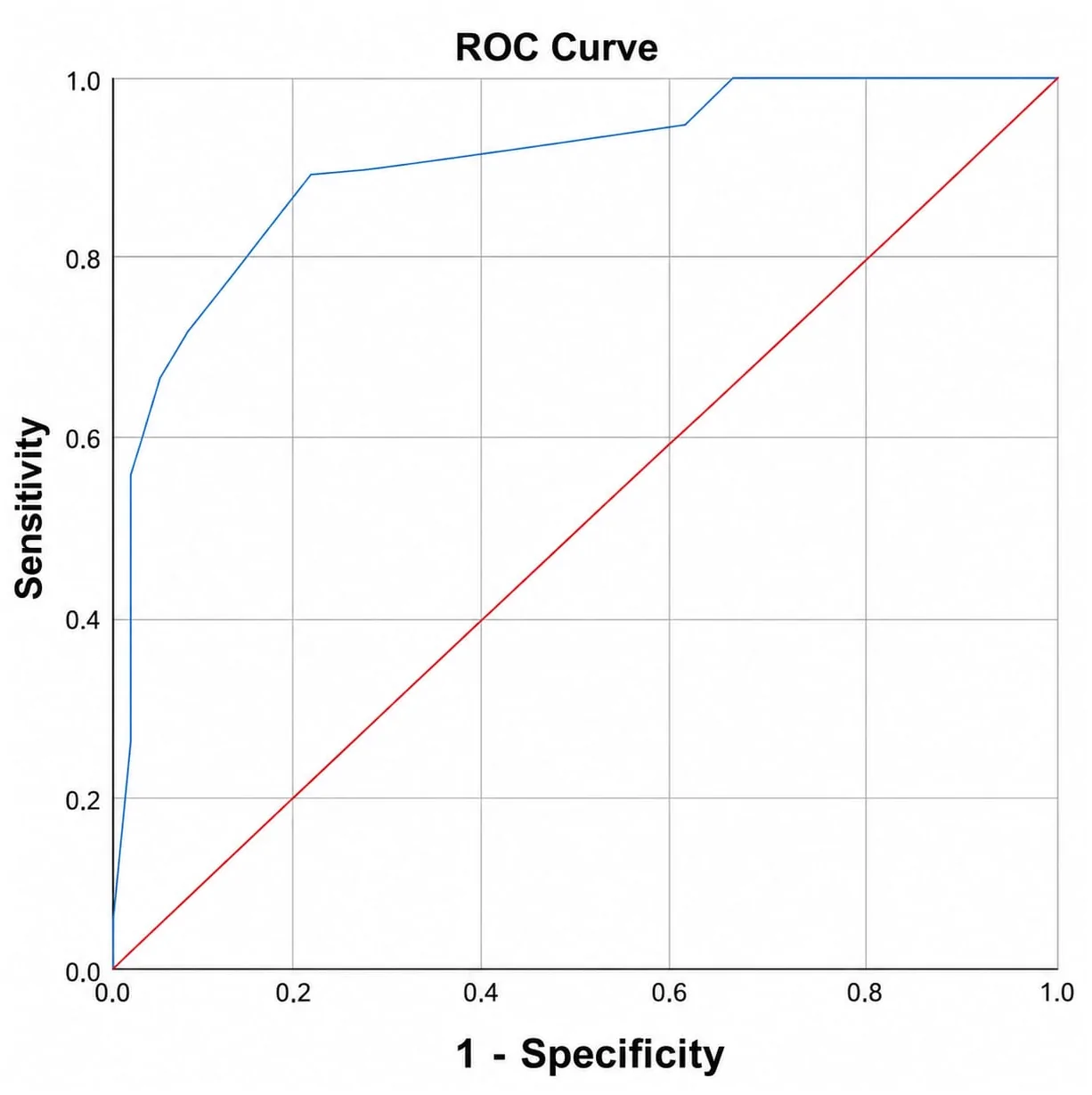
Receiver operating characteristic (ROC) curve illustrating the relationship between sensitivity and 1 − specificity. Receiver operating characteristic (ROC) curve for the prediction of sexual dysfunction in women with gestational trophoblastic disease. The blue curve represents the diagnostic performance of the evaluated model, plotting sensitivity against 1 − specificity across different thresholds. The red diagonal line indicates the reference line corresponding to a classifier with no discriminative ability.

## Discussion

This study identified a predominance of young, married women with secondary education among patients diagnosed with GTD, which is consistent with evidence indicating that GTD primarily affects women of reproductive age. The high proportion of participants identifying as mixed race reflects the ethnic composition of the Brazilian population and underscores the importance of considering racial and social disparities in the management of GTD.

This demographic pattern is consistent with international evidence demonstrating that GTD predominantly affects women of reproductive age. Population-based studies and epidemiological reviews indicate that maternal age remains a major risk factor, with incidence peaking in the second and third decades of life (Braga et al., 2021; Lee et al., 2019). Therefore, our findings reflect the established age-related vulnerability in GTD and support the generalizability of our sample to similar clinical populations.

Hydatidiform mole was the most frequent histopathological diagnosis (87.7%), consistent with the known prevalence of this GTD subtype. No significant association was observed between family history of cancer and sexual dysfunction (*p* = 0.907), although the potential influence of comorbidities warrants further investigation.

Hormonal contraceptive use is essential during GTD follow-up to prevent pregnancy during hCG surveillance. Monitoring adherence is not only clinically necessary but may also help clarify potential effects on sexual health. Caliman et al. (2023) reported that oral contraceptives may influence sexual function in women of reproductive age, highlighting the need for careful evaluation of possible side effects in vulnerable populations.

The interaction between hormonal dynamics and psychological well-being represents a critical aspect of women’s reproductive health, often overlooked in specific clinical conditions such as GTD. Women are at higher risk for anxiety and depressive disorders than men, and fluctuations in sex hormones have been identified as a major biological contributor to this disparity (Kundakovic & Rocks, 2022). In particular, changes in estrogen levels influence brain plasticity and may increase vulnerability to psychopathology (Kundakovic & Rocks, 2022). Thus, hormonal shifts experienced during GTD treatment and recovery may exacerbate psychological vulnerability and, consequently, affect sexual health outcomes.

Fluctuating hormone levels across the menstrual cycle and major reproductive stages have also been associated with mood changes, mental health conditions, and increased risk of psychotic episodes (Wieczorek & Maslowski, 2023). Although hormonal levels were not directly measured in this study, mandatory contraceptive use during GTD follow-up—often involving exogenous hormones—may introduce additional complexity. The impact of these hormonal interventions on mood and sexual function requires further investigation, particularly given evidence that oral contraceptives can influence sexual response (Caliman et al., 2023). This emphasizes the importance of monitoring potential side effects in women recovering from GTD.

Studies of other reproductive conditions, such as polycystic ovary syndrome (PCOS) and infertility, provide relevant comparisons. Women with PCOS have higher rates of depression and anxiety, often associated with hormonal imbalances (Dubé-Zinatelli & Lavoie, 2025). Similarly, infertility—characterized by uncertainty and reproductive loss—has been linked to significant psychological distress (Sharma & Sharma , 2022). These findings underscore how reproductive health conditions, in combination with hormonal fluctuations and psychological stress, can substantially affect women’s mental and sexual well-being. The emotional burden of GTD—characterized by anxiety, depressive symptoms, and fear of recurrence—may predispose women to sexual dysfunction, even when the overall prevalence does not appear to exceed national estimates.

Comparable patterns of psychological vulnerability have been described in other gynecologic disorders. For instance, Abrão et al. (2020) reported that women with endometriosis frequently experience anxiety, depressive symptoms, diminished sexual well-being, and impaired quality of life due to the chronic and emotionally taxing nature of the disease. Although endometriosis and GTD differ considerably in etiology and prognosis, the psychological responses observed by Abrão et al. (2020) parallel the emotional and sexual health challenges reported by participants in the present study.

Considering this broader body of evidence reinforces the importance of addressing mental health and qualityoflife dimensions during the follow-up of women with GTD. Care strategies should extend beyond the immediate physical consequences of the disease and incorporate the complex interplay between hormonal status, psychological well-being, and sexual function. Future studies should further investigate these relationships by including hormonal assessments and comprehensive psychological evaluations to enable more targeted and holistic interventions.

Although no statistically significant difference was observed in the prevalence of sexual dysfunction among women with GTD compared with national estimates (49% among Brazilian women aged 18 yr and 67% among those aged 40–65 yr), larger studies are warranted. Such investigations should account for the clinical and psychosocial complexities associated with GTD management. According to the SQ-F results, most participants reported adequate sexual desire (63.0%), a positive response to foreplay (83.6%), and frequent orgasms (63.0%). However, 15.0% reported dyspareunia, a proportion comparable with national data indicating that approximately 17.8% of Brazilian women experience pain during intercourse.

Women residing in rural areas demonstrated a 3.47-fold higher risk of sexual dysfunction than those living in the metropolitan area of Recife. This disparity may reflect cultural and socioeconomic differences that influence access to healthcare services and support networks, as described by Laumann et al. (2005). Such factors may create additional barriers to the recognition and management of sexual dysfunction, particularly in rural settings. Smoking was also significantly associated with sexual dysfunction (PR = 2.88). Peres (2013) suggested that smoking may compromise genital blood flow and alter sexual response. Although nicotine dependence was not assessed in this study, previous research indicates that greater dependence is associated with reduced sexual desire.

While an increased overall prevalence of sexual dysfunction was not identified, sexual difficulties remain common among Brazilian women. Concerns such as low desire, discomfort, and pain are frequently underreported and inadequately addressed in clinical practice, often due to sociocultural taboos and professional discomfort in discussing sexual health.

The final Poisson regression model demonstrated strong predictive accuracy (AUC = 0.899), supporting its potential utility in identifying women at risk of sexual dysfunction. Nevertheless, several limitations should be acknowledged. The study was based on a small convenience sample and relied on a single instrument (SQ-F) to assess sexual function. Additionally, the absence of a control group of women without GTD limits direct comparisons and precludes definitive attribution of observed dysfunctions solely to the GTD diagnosis or its treatment. Although the findings were compared with national prevalence data, the inclusion of a matched control group would have provided more robust evidence regarding the specific impact of GTD on sexual health. Future studies should prioritize the incorporation of such comparison groups to strengthen causal inferences and to clarify the unique challenges faced by women with GTD.

Despite these limitations, this study advances current understanding of the potential effects of GTD on women’s sexual health. It underscores the importance of adopting a multidisciplinary, patient-centered model to care that considers sociocultural determinants—such as stigma, religiosity, and educational level—which may influence both the experience and reporting of sexual dysfunction.

The absence of a formal comparison group without GTD remains a recognized methodological limitation. Nevertheless, situating the findings within prevalence data from Brazilian female populations provides important context. Previous studies have reported sexual dysfunction prevalence rates ranging from approximately 49%–51% (Abdo et al., 2004) to 25%–63% among women aged ≥18 yr (Abdo , 2009). In contrast, 63.0% of participants in the present study reported being sexually satisfied, a proportion that may initially appear high. However, self-reported satisfaction should be interpreted cautiously, as sexual dysfunction may affect specific domains without necessarily resulting in overall dissatisfaction. Direct comparisons with general population data are inherently limited due to the distinct psychosocial and hormonal context associated with GTD. Nevertheless, the prevalence of specific dysfunctions observed in this cohort—such as dyspareunia (15.0%) and difficulty with concentrating during sexual activity (45.2%)—can be considered alongside domain-specific findings from studies of Brazilian women. Although not a substitute for a matched control group, these reference data broaden interpretation and highlight the need for future controlled investigations.

## Conclusions

This study did not demonstrate a significantly higher prevalence of sexual dysfunction among women with GTD receiving outpatient care compared with national estimates for Brazilian women. However, residence in rural areas of Pernambuco and smoking were associated with an increased risk of sexual dysfunction compared with residence in metropolitan areas and nonsmoking status.

In clinical practice, healthcare professionals should be adequately trained to address female sexuality with sensitivity and openness, thereby overcoming persistent sociocultural taboos surrounding the topic. The incorporation of validated assessment tools, such as the SQ-F, may facilitate early identification of dysfunction and support the implementation of individualized, multidisciplinary interventions. As a fundamental component of overall quality of life, sexual health should be systematically integrated into the outpatient management of women with GTD to promote comprehensive biopsychosocial well-being during and after treatment.

### Future directions

Further research is required to deepen understanding of the multidimensional aspects of sexual health in women with GTD. Future studies should include appropriately matched control groups to enable more rigorous comparative analyses and to identify population-specific risk markers.

Larger and more diverse samples representing different geographic and sociocultural contexts are recommended to enhance the generalizability of findings across Brazil. The integration of quantitative and qualitative methodologies may yield richer insights into women’s subjective experiences, including their perceptions, emotions, and expectations regarding sexuality during and after GTD treatment.

Most available studies focus on short-term outcomes, limiting insight into the long-term consequences of GTD and its management on sexual function and quality of life. Longitudinal research is therefore strongly recommended to monitor patients over time and to evaluate sustained effects.

Multicenter investigations involving varied healthcare settings and regions are also needed to improve representativeness and comparability. Given the high likelihood of underreporting—particularly in sociocultural contexts characterized by stigma and silence—qualitative studies that center on patients’ perspectives may provide valuable contributions to the development of more sensitive, inclusive, and integrated models of care.

##  Supplemental Information

10.7717/peerj.21427/supp-1 Supplemental Information 1 SPSS code used to calculate questionnaire scores included in the dataset and used in the statistical analyses of sexual dysfunction in women with gestational trophoblastic disease SPSS syntax used to compute questionnaire scores prior to statistical analysis.

10.7717/peerj.21427/supp-2 Supplemental Information 2 SPSS code used to fit the Poisson regression model to estimate associations between clinical and sociodemographic variables and sexual dysfunction in women with gestational trophoblastic disease SPSS code used to fit the Poisson regression model for the analysis of factors associated with sexual dysfunction in women with gestational trophoblastic disease.

10.7717/peerj.21427/supp-3 Supplemental Information 3 Dataset containing sociodemographic, clinical and questionnaire variables used to analyze sexual dysfunction in women with gestational trophoblastic disease in Recife, Pernambuco, Brazil

10.7717/peerj.21427/supp-4 Supplemental Information 4 STROBE checklist

## Additional Information and Declarations

### Data Availability

The following information was supplied regarding data availability: The raw data are available in the Supplemental Files.
