# Supplementary material for: Sexual dysfunction in women with gestational trophoblastic disease: a cross-sectional study in Recife, Pernambuco, Brazil
Source: PeerJ. 2026 Sep 25;14:e21427. doi: 10.7717/peerj.21427 (PMC13618446; doi:10.7717/peerj.21427)
Supplement: Supplementary file 1 [file peerj-14-21427-s004.docx]

**STROBE Statement—Checklist of items that should be included in reports of cross-sectional studies**

| **Section/Topic** | **Item No** | **Recommendation** | **Reported on page/section** |
| --- | --- | --- | --- |
| **Title and abstract** | 1 | Indicate the study’s design with a commonly used term in the title or abstract. | Title page, Abstract |
|  | 2 | Provide in the abstract an informative and balanced summary of what was done and what was found. | Abstract |
| **Introduction** | 3 | Explain the scientific background and rationale for the investigation. | Introduction |
|  | 4 | State specific objectives, including any prespecified hypotheses. | End of Introduction |
| **Methods** | 5 | Present key elements of study design early in the paper. | Start of Methods |
|  | 6 | Describe the setting, locations, and relevant dates. | Study Design and Setting |
|  | 7 | Give the eligibility criteria, and the sources and methods of selection of participants. | Participants section |
|  | 8 | Clearly define all outcomes, exposures, predictors, potential confounders, and effect modifiers. | Data Collection section |
|  | 9 | Describe any efforts to address potential sources of bias. | Discussion - Limitations |
|  | 10 | Explain how the study size was arrived at. | Sample section |
|  | 11 | Explain how quantitative variables were handled in the analyses. | Data Analysis section |
|  | 12 | Describe all statistical methods, including those used to control for confounding. | Data Analysis section |
| **Results** | 13 | Report numbers of individuals at each stage of study. | Results |
|  | 14 | Give characteristics of study participants and information on exposures and potential confounders. | Results - Tables 1 to 4 |
|  | 15 | Report numbers of outcome events or summary measures. | Results - Table 5 |
|  | 16 | Give unadjusted estimates and confounder-adjusted estimates. | Results - Tables 6 to 8 |
|  | 17 | Report other analyses done (e.g., sensitivity or subgroup analyses). | Results - Final Poisson regression model |
| **Discussion** | 18 | Summarize key results with reference to study objectives. | Discussion - First paragraph |
|  | 19 | Discuss limitations of the study. | Discussion - Limitations paragraph |
|  | 20 | Give a cautious overall interpretation of results considering objectives, limitations, multiplicity of analyses, results from similar studies, and other relevant evidence. | Discussion - Final paragraphs |
|  | 21 | Discuss the generalizability (external validity) of the study results. | Discussion - Generalization paragraph |
| **Other information** | 22 | Give the source of funding and the role of the funders. | **No funding was received for this study.** |
